# Is “Delayed Onset Muscle Soreness” a False Friend? The Potential Implication of the Fascial Connective Tissue in Post-Exercise Discomfort

**DOI:** 10.3390/ijms22179482

**Published:** 2021-08-31

**Authors:** Jan Wilke, Michael Behringer

**Affiliations:** 1 Department of Sports Medicine and Exercise Physiology, Institute of Sports Sciences, Goethe University, 60487 Frankfurt am Main, Germany; behringer@sport.uni-frankfurt.de; 2 Division of Exercise, Health & Performance, Institute of Occupational, Social and Environmental Medicine, Goethe University, 60590 Frankfurt am Main, Germany

**Keywords:** DOMS, fascia, eccentric exercise, pain, athletes

## Abstract

Strenuous and unaccustomed exercise frequently lead to what has been coined “delayed onset muscle soreness” (DOMS). As implied by this term, it has been proposed that the associated pain and stiffness stem from micro-lesions, inflammation, or metabolite accumulation within the skeletal muscle. However, recent research points towards a strong involvement of the connective tissue. First, according to anatomical studies, the deep fascia displays an intimate structural relationship with the underlying skeletal muscle and may therefore be damaged during excessive loading. Second, histological and experimental studies suggest a rich supply of algogenic nociceptors whose stimulation evokes stronger pain responses than muscle irritation. Taken together, the findings support the hypothesis that DOMS originates in the muscle-associated connective tissue rather than in the muscle itself. Sports and fitness professionals designing exercise programs should hence consider fascia-oriented methods and techniques (e.g., foam rolling, collagen supplementation) when aiming to treat or prevent DOMS.

## 1. Introduction

Delayed onset muscle soreness (DOMS) is a frequent post-exercise phenomenon that typically manifests after strenuous and unaccustomed loading. Its symptoms, comprising pain and stiffness of the affected soft tissue, occur hours following activity, reaching maximal levels after one to three days [1]. While all types of muscle work may induce general muscular exhaustion, DOMS is particularly caused by eccentric contraction [1,2]. Related examples include downhill running [3], ballistic stretching [4], plyometrics [5], and eccentric resistance exercise [1]. Multiple theories exist attempting to explain the mechanisms leading to DOMS and most of them suggest an important role of the skeletal muscle (e.g., excessive lactate production, inflammation, structural damage). Yet, recent research suggests a substantial mechanical and/or sensory contribution of the extramuscular connective tissue. As the optimal stimuli needed to trigger adaptations of the deep fascia are substantially different to those for the skeletal muscle [6], this may have significant implications with regard to the prevention and treatment of DOMS. The present article therefore reviews the available evidence describing the possible involvement of the deep fascia in DOMS.

## 2. Classical Pathogenetic Models

One of the oldest theories describing the possible mechanism of DOMS focuses on the accumulation of lactic acid. Lactate is a substance produced during glycolytic metabolism and has been reported to induce pain when injected into skeletal muscles with [7,8] or without the addition of ATP or protons [9]. Excessive lactate concentrations may therefore represent a noxious stimulus for free nerve endings of type III [9] and IV [10] afferents, which are located within small blood vessels of skeletal muscles. Being connected to the central nervous system via thinly myelinated (type III) or unmyelinated (type IV) nerve fibers [11], the free or unencapsulated nerve endings act as the major nociceptors of the skeletal muscles and respond to mechanical and chemical stimuli [12]. Although there is evidence suggesting that receptor activation by metabolites may be involved to some extent in muscular fatigue during intense exercise [13], the lactic acid theory has been largely rejected as a cause of DOMS [1]. One reason for this is the time component. Lactate concentrations promptly return to the baseline after cessation of physical activity, whereas the DOMS-typical discomfort occurs with a delay. A second counter-argument is the lacking correlation between lactate concentrations and the strength of DOMS. Although concentric exercise does only very rarely lead to DOMS, lactate production is considerably higher than in eccentric exercise even if the power output is matched [14]. This observation fits with data showing high lactate concentrations and no DOMS after level running but low lactate levels and substantial DOMS after downhill running [15].

Almost 120 years ago, Hough [16] first proposed the theory that DOMS could result from structural damage within the skeletal muscle. It has repeatedly been claimed that high mechanical stresses imposed to the soft tissue could exceed the load-bearing capacity of the sarcomere, leading to micro-ruptures located in or near the Z-disk (e.g., [17,18]). In fact, such changes have regularly been identified during microscopic examinations of biopsies taken after unaccustomed eccentric contractions [19,20]. The Z-line streaming more frequently affects type II fast-twitch muscle fibers than type I slow-twitch muscle fibers [1]. As insinuated by their names, fast-twitch fibers exhibit a high force, power, and speed potential at limited endurance, while slow-twitch fibers achieve low contraction speeds but high fatigue resistance. Compared with type I fibers, the fast-twitch type II fibers have narrower and weaker Z-disks and less compliant titin filaments, which could lead to greater mechanical stresses during eccentric contraction [1,21,22]. Furthermore, they contain smaller isoforms of the sarcomere-stabilizing protein nebulin, potentially leading to increased susceptibility to strain-induced damage [23]. All these factors, acting in concert, probably explain why muscles with a large type II fiber content sustain greater damage from eccentric contraction than those with a larger type I fiber content [24]. However, some authors interpret the microscopic intracellular structural changes following eccentric contraction as a natural part of the remodeling process rather than a sign of muscular damage [25]. Furthermore, it remains unclear how this micro-traumatization should be associated with pain. Of course, an activation of mechanosensitive type III afferents due to structural lesions is conceivable, but this could only explain current pain sensations but not a delayed onset and the persistence of pain after exercise. Finally, the activation threshold of these receptors is very high, which prevents activation through normal contractions. The assumption that morphological muscle damage is associated with DOMS symptoms also lacks clinical and experimental support. Nosaka et al. [26] instructed male participants to perform eccentric exercise bouts with variable repetition numbers. While induction of DOMS was successful, pain experienced upon movement and palpation did not correlate with plasma creatine kinase, which is a marker of muscle damage. Similar findings had been made by Nurenberg et al. [27] who found only a weak correlation between the DOMS grade and the degree of ultrastructural tissue injury as analyzed with electron micrographs.

A third complex of theories focuses on inflammatory processes. Contrarily to tissue damage, a delayed and sustained activation of type-III afferents may be induced via inflammation-associated intramuscular swelling, which exerts pressure on the mechanosensitive receptors. This is supported by the observation that maximal swelling coincides with the post-exercise DOMS peak [28]. In addition, inflammation could lead to increased pain perception via the release of pain-modulating substances (such as PGE2) by inflammatory cells invading the damaged muscle tissue. It is assumed that the inflammatory cells, which enter the damaged tissue, are chemotactically guided to the site via substances that diffuse into the plasma and interstitial space [29]. Chemotaxis describes the movement of immune cells towards substances (attractants) that are released from damaged cells. One example of a chemotactically active substance is the chemoattractant protein MCP-1. It is highly expressed in injured muscle cells and attracts macrophages [30]. Interestingly, there is a possible link between the inflammation and the previously mentioned muscle damage theory [31]. High mechanical loads do not only seem to cause damage to the contractile elements of the muscle fibers but also to the membranes. The resulting change in membrane permeability could be the basis for the release of chemotactically active substances.

The above assumptions fit with Armstrong’s early theory of the mechanisms underlying DOMS [15,32]. He hypothesized that membrane damage resulting from high mechanical forces results in an uncontrolled influx of calcium from the interstitial space, which activates calcium-dependent proteases and phospholipases that further exacerbate membrane damage. According to his theory, this process is amplified by the fact that calcium accumulates within the mitochondria, affecting cellular respiration and thus ATP synthesis and ultimately reducing the active return of calcium into the sarcoplasmic reticulum by SERCA pumps. The membrane damage further results in an efflux of intracellular components, such as enzymes, into the intercellular space and thereby attracts monocytes that convert to macrophages. Mast cells and histocytes are activated and the accumulation of chemicals resulting from phagocytosis and necrosis are then thought to activate free nerve endings that trigger the pain. However, some authors have challenged the existence of an exercise-induced sarcolemma injury or inflammation, as no sarcolemma injury could be found after eccentric contractions despite the presence of severe DOMS [33]. This is supported by others who only found very weak [26] or no evidence [34] of inflammation and necrosis in muscles following exercise-induced muscle damage. Some authors even speculate that the observed inflammation in human skeletal muscle after exercise may rather represent a methodological artefact than a true exercise-induced event, due to the invasive nature of muscle sampling [34].

Finally, free radicals are also repeatedly cited as a reason for damage to the muscle fiber membrane [35,36]. The fact that they are produced during eccentric contraction is not controversial as numerous studies have shown this connection so far [37,38]. However, it is unclear as to whether the free radicals are causally linked to the development of exercise-induced membrane damage and DOMS. Among other factors, the time component speaks against such a connection. Close et al. [39] demonstrated that the increase in free radicals occurred only 24–48 h after maximal DOMS. In addition, the administration of antioxidants had little or no effect on soreness. This is supported by De Oliveira and colleagues [40] who found an antioxidant vitamin supplementation to prevent oxidative stress but not DOMS. Some authors now even assume that the free radicals formed rather have a physiological signaling effect and should be interpreted less in terms of a pathological influence [39]. Finally, as pointed out by Close et al. [35], it should be noted that the mitochondrial production of ROS is highly associated with increases in aerobic metabolism. It is assumed that ROS are produced at a rate of 0.15% of oxygen consumption [41]. Therefore, one would expect that exercise associated with high oxygen consumption rather than eccentric contractions would be associated with increased DOMS.

In summary, the classical pathogenic models focusing on the muscle tissue are not able to explain the symptoms of DOMS commonly observed after unaccustomed eccentric contractions.

## 3. Possible Involvement of the Connective Tissue

**Figure 1:**
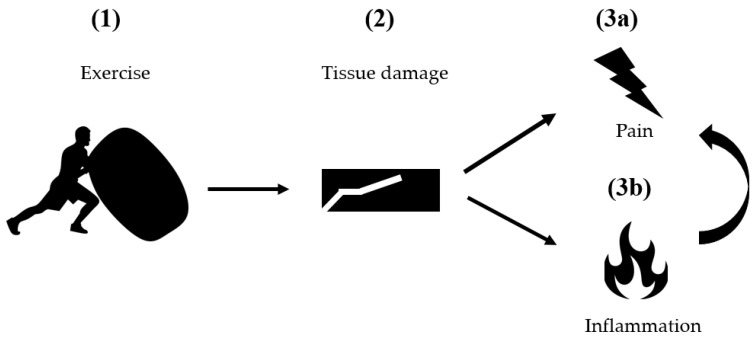
Schematic model of a fascial DOMS-origin. During strenuous exercise (**1**), morphological damage occurs in the extramuscular connective tissue (**2**), which stimulates algogenic free nerve endings (**3a**). At the same time, local inflammation and edema produce local swelling and further increase pain (**3b**). Original figure created by the authors.

A recent stream of research suggests that the collagenous connective tissue could represent the pathogenic substrate of DOMS. Based on the available evidence, both the architectural features and the sensory capacity of fascia may be causal for the perceived post-exercise discomfort (for a conceptual illustration, see Figure 1).

### 3.1. Structural Damage: Anatomy of Fascia

If not dissected in cadavers or damaged during surgery, the collagenous connective tissue is inextricably linked to the skeletal muscle. On the micro-level, the muscle fiber fuses tightly with the endomysium [42]. Interestingly, up to 70% of the muscle fibers, do not span the complete distance between insertion and origin [43]. Yet, as the endomysia of adjacent fibers are structurally connected, they can still transmit force through translaminar shearing [43]. Similar to the endomysium, the perimysium also does not only represent an envelope for muscle fiber bundles. Rather, it forms a honeycomb-like network of collagenous tubes with direct tissue continuity [44]. This has practical implications because the perimysial web has been demonstrated to transmit a radial force upon mechanical loading [44]. Finally, the deep fascia has been shown to exhibit direct fibrous expansions merging with the underlying skeletal muscle, which means that muscular contraction can selectively tension it [45]. Besides being structurally connected to the underlying muscle, fascia does also provide a direct linkage to other muscles arranged parallel (e.g., from the tibialis anterior to the extensor digitorum) or in-series (e.g., from the gastrocnemius to the hamstrings) [46]. It is therefore tenable to assume that any muscular activity or loading will have pronounced mechanical effects on the connective tissue.

As outlined, DOMS primarily occurs during eccentric loading, and during this active lengthening, high strain forces act upon the skeletal muscle. From a functional point of view, the described continuities to the collagenous soft tissue may represent a shock absorber taking up excessive forces potentially damaging the skeletal muscle [46,47]. However, if going beyond the loading capacity, microscopic or macroscopic damage may occur in the endomysium, perimysium, or deep fascia. Following this paradigm, some studies have examined the morphology and integrity of the collagenous connective tissue during loading or following induction of DOMS.

### 3.2. DOMS-Specific Evidence

Despite its denomination, muscle injuries rarely affect only the muscle tissue [47]. In about 90% of the cases, the actual site of injury is either located in the musculotendinous junction or in the extramuscular fascia [47]. Like DOMS, muscle injury frequently occurs after active excessive eccentric loading, and therefore both may exhibit a similar involvement of the connective tissue. Interestingly, there is compelling evidence for the existence of structural damage of the extracellular matrix in DOMS. Brown et al. [48] instructed their participants to perform 50 maximal eccentric contractions of the knee extensors. Increased urinary hydroxyproline and hydroxylysine levels, suggestive of connective tissue breakdown, were measured immediately as well as two days post-activity. In a more recent experiment, at 24 to 72 h after induction of DOMS, Mavropilas et al. [49] found hydroxyproline elevations amounting up to 53%. Yet, interestingly, similar to the lacking correlation with markers of muscle damage, the observed changes were not associated with the perceived DOMS magnitude. Additional research is hence urgently warranted to clarify the relation between structural connective tissue damage and DOMS-related pain.

Raastad et al. [50] examined tissue biopsies sampled from the vastus lateralis muscle of healthy volunteers performing a total of 300 maximal eccentric contractions of the knee extensors. On the days following exercise, immunoreactivity for tenascin-C and N-terminal propeptide of procollagen type III, two markers of extracellular matrix remodeling, was strongly increased. In eight untrained males, Crameri et al. [51] induced DOMS using electrostimulation in one leg and active eccentric contraction (isokinetic dynamometer) in the other leg. Electrostimulation caused significant degrees of intracellular disruption, Z-disk destruction, and satellite cell markers. Surprisingly, these observations were only rarely made after active contraction. While this finding raises questions about the relevance of muscular damage, increased staining for tenascin-C was observed in both conditions, with two individuals displaying a surge after as little as five hours.

In a recent experiment, Tenberg et al. [52] examined fascial morphology and mechanics following exhausting eccentric and concentric exercise of the elbow flexors. Contrarily to concentric loading, which does not lead to DOMS, brachial fascia thickness increased on the days after eccentric exercise. Importantly, fascial thickening correlated with subjective DOMS pain upon palpation, suggesting a clinical relevance of the observation. Besides an accumulation of hyaluronic acid, the thickness increase in the fascia could particularly reflect edema and inflammation resulting from fascial tissue injury. Changes were also seen in tissue mobility. While muscle displacement during passive joint movement was higher after eccentrics, fascial mobility remained unchanged. Additional research is warranted in order to judge whether an inability of the fascia to follow movement of the muscle has a pathogenic value in DOMS. In sum, the available evidence suggests the existence of morphological changes in the extramuscular connective, some of which are directly linked to subjective discomfort.

### 3.3. Sensory Contribution: Physiology of Fascia

Fascia is suggested to substantially contribute to proprioception and pain owing to its rich equipment with sensory receptors. Specifically, histological analyses confirmed the existence of Ruffini corpuscles [53,54], Pacini corpuscles [53,54], and free nerve endings [53,55,56,57,58]. As a substantial portion of the identified fibers have been reported to be positive for substance P and the calcitonin gene-related peptide (CGRP), an algogenic capacity of at least some free nerve endings must be assumed [55,56,57,58]. Barry et al. [57] compared the nerve fiber density of different lumbar tissues in rodents, reporting a threefold higher presence in the fascia as compared to the muscle.

Owing to its sensory innervation, fascia has been suggested to represent a clinically relevant potential pain generator. Taguchi et al. [56] applied mechanical, chemical, and thermal stimuli to the rat crural fascia. Repetitive pinching with a sharpened watchmaker’s forceps increased production of c-FOS, a gene expressed in neurons, which reflects pain-related neural activation. Irritating the fascia by means of a cotton ball soaked with bradykinin led to slowly increasing discharge rates in 13 out of 23 tested c-fibers carrying sensory (e.g., nociceptive) information. While only a few fibers responded to cold application, heat elicited activity of more than half of the c-fibers. In another experiment, Schilder et al. [59] injected hypertonic saline solution into the subcutaneous tissue, the deep fascia, and the muscles of the lower back region. Chemical irritation of the non-muscular structures provoked more sustainable (~15 vs. ~10 min) and stronger pain sensations than muscular injection. Additionally, only after fascial irritation, participants used affective pain descriptors (e.g., agonizing, heavy, and killing), which are frequently related to reports of low back pain. Deising et al. [60] performed a similar study stimulating different lumbar tissues by means of the nerve growth factor (NGF), whose injection evokes local hyperalgesia. Following the experiment, long-lasting sensitizations to mechanical and chemical stimulation (up to two weeks) were observed with regard to the fascia. This is of interest because DOMS complaints can persist for several days [1].

The higher sensitivity of fascia to noxious stimuli does not only apply to chemical agents. Again targeting the lower back region, Schilder et al. [61] showed that the same response pattern (stronger pain sensation upon fascial vs. muscular irritation) occurs in electrical stimulation. In addition to exhibiting a high general pain sensitivity, fascia seems to respond strongly to local inflammation. Hoheisel & Mense [62] injected Freund’s complete adjuvant in the rat thoracolumbar fascia, causing an inflammatory response. Interestingly, 11% of neurons at the L3 level, which normally do not respond to input from the lumbar fascia, became active. Furthermore, some nerve cells displayed new receptive fields.

Taken together, the available evidence suggests that the fascial connective tissue rather than the muscle may play a role in the development and perception of soft tissue pain.

### 3.4. DOMS-Related Evidence

Gibson et al. [63] instructed a sample of 13 healthy young adults to perform an eccentric loading protocol for the tibialis anterior muscle of one leg. One day after induction of DOMS, hypertonic saline was injected into the tibialis anterior muscle and its deep fascia. In the non-exercised control leg, irritation of the fascia was more painful (+42%) than stimulation of muscle tissue, which accords with the above findings of Schilder and colleagues [59]. The same pattern (higher pain upon fascial injection) was found in the exercised leg, however, there was a marked difference. While pain following muscular irritation was identical to the control leg, stimulation of the fascia was 39% more painful. This means that the deep fascia, but not the skeletal muscle, becomes more sensitive to noxious chemical stimuli in the presence of DOMS. In the study of Lau et al. [64], ten young male participants performed 10 × 6 maximal isokinetic eccentric contractions of the non-dominant elbow flexors in order to induce DOMS. On the following days, the authors measured the electrical pain threshold using ultrasound imaging and needle electrodes. In line with previous findings, pain intensity changes from the baseline were higher for the biceps brachii and the brachialis fascia as compared to the skeletal muscle at 48 h post-exercise. Whilst changes in fascial sensitivity upon electrical stimulation were not associated with palpation pain, a moderate to strong correlation existed with the mechanical pain threshold (r = 0.63–0.87).

## 4. Practical Implications and Perspectives for Future Research

**Figure 2:**
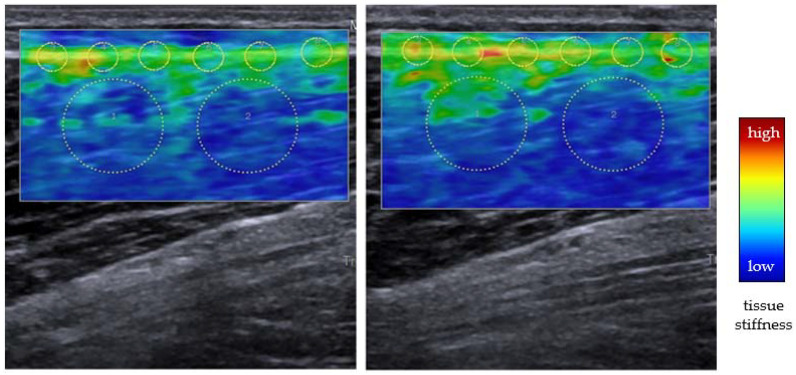
Shear-wave elastography (SWE) of the ventral thigh before (**left**) and 48 h (**right**) after lower leg eccentric exercise. In SWE, an acoustic radiation force impulse is used to produce shear waves traveling perpendicularly. Measuring the speed of this horizontal shear wave propagation allows the calculation of mechanical properties. The colored regions of interest indicate the tissue’s mechanical stiffness: while blue represents low values, red represents high values. Note the marked stiffness increase in and near the deep fascia over the muscle (small circles). The scans used for this figure were acquired in the authors’ laboratory.

Hitherto, the majority of the available research on DOMS has been dedicated to the study of the skeletal muscles. For instance, shear wave elastography represents a promising and straightforward method to quantify the mechanical stiffness of the soft tissue. With regard to DOMS, its application is particularly interesting because it allows a clear differentiation assessment of structures (i.e., muscle vs. fascia). Agten et al. [65] examined a sample of 10 volunteers on the days following heavy eccentric exercise of the elbow flexors. Shear wave velocity (a surrogate of mechanical stiffness), measured exclusively inside the muscles, increased immediately post-exercise but returned to the baseline around day two. However, as subjective pain levels reached their peak on day three, a causal relationship with DOMS seems improbable. In view of this and considering the accumulating evidence on the potential implication of the fascia, future studies should use imaging-based methods to study morphological and mechanical changes (i.e., stiffness and elasticitiy) of the deep fascia (Figure 2).

Besides helping to clarify the pathophysiology underlying DOMS, focusing on the collagenous connective tissue could open new avenues for its treatment. According to a systematic review with meta-analysis from Dupuy et al. [66], active recovery, massage, the use of compression garments, immersion, contrast water therapy, and cryotherapy are effective in improving DOMS complaints. Assuming the presence of structural damage in the deep fascia of the skeletal muscle, the timely supply of building material for tissue repair should be crucial for recovery. The oral administration of gelatin has been shown to increase the concentration of the amino acids: glycine, proline, hydroxyproline and hydroxilysine, which are of essential importance for collagen production [67]. In addition, gelatin intake caused a twofold increase of the N-terminal peptide of pro-collagen I (PINP), which indicates stronger collagen production [67]. In a double-blind randomized, controlled trial [68], 24 recreationally active male adults received a placebo or 20 g collagen peptides daily over the period of one week before until 48 h after a fatigue protocol with 150 drop jumps. At two days post-exercise, the DOMS magnitude was substantially lower in the verum group (90.4 vs. 125.7 mm on a visual analogue scale, d = 2.6) as compared to the placebo group. Additionally, individuals with gelatin supplementation restored decrements in counter-movement jump height (a measure of lower leg explosive force) faster than participants with placebo intake (90% vs. 79% of baseline performance at day two). In future trials, it should be evaluated if chronic collagen supplementation can prevent further reduce DOMS and if different doses can modify the effect observed in this initial exploratory trial.

Foam rolling, an intensive form of self-massage with polypropylene tools, represents another promising method to address fascial alterations after strenuous exercise. Krause et al. [69] demonstrated that an acute bout of rolling in the thigh reduces pain sensitivity while improving the relative sliding capacities of the individual fascial layers. Altered intrafascial gliding, which is normally ensured owing to the presence of hyaluronic acid, has been linked with the occurrence of pain [70]. Possibly, the improvement of intrafascial sliding can explain why rolling decreases soft tissue pain [71,72] and DOMS [72,73,74]. Additional research may combine specific assessments of fascial and muscular tissue components (i.e., using ultrasound and elastography imaging, see Figure 2) as well as subjective pain ratings after rolling treatments.

While both collagen supplementation and foam rolling represent relatively passive strategies to trigger adaptations of the soft tissue, conditioning coaches and health professionals may consider designing specific exercise paradigms aiming to increase the tolerance of fascia to loaded lengthening. Besides regular eccentric training and plyometrics, this may particularly include multidirectional dynamic stretching at varying velocities. However, although the connective tissue has generally been demonstrated to substantially adapt to mechanical stimuli [6], studies specifically addressing the impact of exercise on the morphology and mechanics of the deep fascia are still scarce.

## 5. Conclusions

Although theories explaining the pathogenesis of DOMS have long focused on the skeletal muscle, there is accumulating evidence suggesting a prominent role of the collagenous connective tissue. Based on the available literature, strain forces associated with eccentric contraction may cause micro-ruptures and inflammation of the deep fascia. As experimental research clearly demonstrates that fascia is more pain-sensitive than muscle following chemical, thermal, electrical, and mechanical irritation, we propose that delayed onset soft tissue stiffness (DOSS) is a more precise descriptor of the post-exercise phenomenon. Currently, there is still a lack of studies examining fascia-specific approaches for the prevention and treatment of DOSS. However, initial evidence suggests that foam rolling and supplementation with collagen peptides may represent promising options to alleviate the post-exercise-discomfort. Sports and fitness professionals may hence modify previous approaches designed to prevent and treat DOSS, now providing more specific stimuli for the deep fascia.

## Data Availability

Not applicable.
